# Breakage and retention of thoracic paravertebral catheter: a case report

**DOI:** 10.1186/s40981-016-0074-1

**Published:** 2017-01-05

**Authors:** Tasuku Fujii, Yasuyuki Shibata, Kimitoshi Nishiwaki

**Affiliations:** 1 Department of Anesthesiology, Nagoya University Hospital, 65 Tsurumai-cho, Showa-ku, Nagoya, 466-8550 Japan; 2 Department of Surgical Center, Nagoya University Hospital, 65 Tsurumai-cho, Showa-ku, Nagoya, 466-8550 Japan; 3 Department of Anesthesiology, Nagoya University Graduate School of Medicine, 65 Tsurumai-cho, Showa-ku, Nagoya, 466-8550 Japan

**Keywords:** Thoracic paravertebral block, Paravertebral catheter, Breakage, Retention, Complication

## Abstract

**Background:**

Paravertebral catheters are generally inserted and removed without complications. However, catheter breakage occurs rarely. This is the first report describing breakage of a thoracic paravertebral catheter and retention of the catheter fragment within the patient.

**Case presentation:**

A 65-year-old female patient complained of an unusual sensation in her back during postoperative chemotherapy for lung cancer. A catheter fragment was identified in the soft tissues of the back on computed tomography. The paravertebral catheter had been placed 2 years prior left lower lobectomy. The patient had neither neurological symptoms nor infection signs around the fragment. However, the potential side effects of chemotherapy, including coagulopathy and immunosuppression, increased the risk of late-onset hematoma and abscess formation around the fragment. Therefore, we surgically removed the catheter fragment. Analysis of the fragment revealed that the catheter had been severed by the cutting edge of the Tuohy needle or the suture needle.

**Conclusion:**

In this report, a paravertebral catheter fragment was retained in the posterior mediastinum for 2 years. The catheter was likely damaged during the insertion procedure. We suggest that catheters should not be withdrawn through the Tuohy needle, but be withdrawn together with the Tuohy needle. Although secure fixation of the catheter can be achieved with sutures, to reduce the risk of damage to the catheter, alternative methods, such as surgical tapes or skin glue should be considered. After removal of a catheter, its tip should be checked to ensure that the entire catheter has been completely removed. If a catheter fragment is retained within the patient, removal of the fragment should be considered according to the patient’s condition and risks.

## Background

Thoracic paravertebral block (TPVB) is an effective alternative to epidural anesthesia for the management of perioperative pain in thoracotomy [1]. Epidural anesthesia and TPVB are generally performed without complications. However, a catheter is rarely broken or sheared, during insertion or removal procedure. Although breakage of epidural catheters has been previously reported [2–5], there have been no reports of this complication with paravertebral catheters. In this report, we present the first case of breakage of the paravertebral catheter, and retention of the catheter fragment within the patient.

## Case presentation

A 65-year-old female patient (American Society of Anesthesiologists physical status II; height, 146 cm; weight, 44.2 kg) complained of an uncomfortable sensation in her back during postoperative chemotherapy for lung cancer. The only physical sign was slight focal tenderness on palpation, without evidence of inflammation. Blood test results were normal.

The patient had a history of lung cancer, and had undergone left lower lobectomy at our hospital 2 years prior to this incident. Before the operation, ultrasound-guided TPVB was performed under general anesthesia, with the patient in a lateral decubitus position. With the aid of a portable ultrasound machine (M-Turbo®; FUJIFILM SonoSite, Inc., Bothell, WA), an 18-gauge Tuohy needle (Epidural anesthesia set; Hakko, Nagano, Japan) was inserted from the outer end of the ultrasonic linear array transducer via the fifth intercostal space. The needle was advanced in-plane with the transducer in a lateral-to-medial direction. After the needle tip had been reached into the thoracic paravertebral space, 5–10 mL of saline was injected via the needle, and expansion of the thoracic paravertebral space was confirmed by ultrasonography. A radiopaque catheter (1.0 × 950 mm polytetrafluoroethylene catheter; Hakko, Nagano, Japan) was inserted to a depth of 5 cm into the thoracic paravertebral space through the Tuohy needle. The catheter was fixed to the skin with 4-0 nylon sutures. For perioperative pain management, 0.5% ropivacaine (20 mL) was injected via the paravertebral catheter prior to the surgical procedure. At the conclusion of surgery, the patient received a bolus injection of 0.5% ropivacaine (10 mL), followed by a continuous infusion of 0.4% ropivacaine at 6 mL/h, via the paravertebral catheter. Intravenous administration of a nonsteroidal anti-inflammatory drug (NSAIDs) (flurbiprofen) and intravenous patient-controlled analgesia (iv-PCA) of morphine (bolus dose, 1 mg; lockout interval, 10 min) were used for rescue postoperative analgesia. Flurbiprofen (50 mg) was administered intravenously 1 h after surgery. A total of 18 mg of morphine was used during the 24-h period after surgery. On postoperative day one, the continuous TPVB infusion was discontinued, and the paravertebral catheter removed by the patient’s attending physician. Leakage of local anesthetic from the catheter insertion site was not observed.

**Fig. 1:**
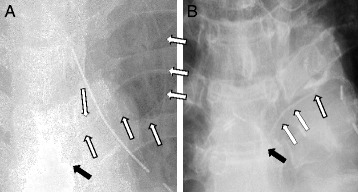
Chest radiographs showing the position of the catheter after insertion and the retained catheter fragment. The *left* image (**a**) shows the paravertebral catheter after the insertion procedure (*white arrows*). The right image (**b**) shows the catheter fragment (*white arrows*) remaining after catheter removal. In both images, the *black arrow* indicates the tip of the catheter

**Fig. 2:**
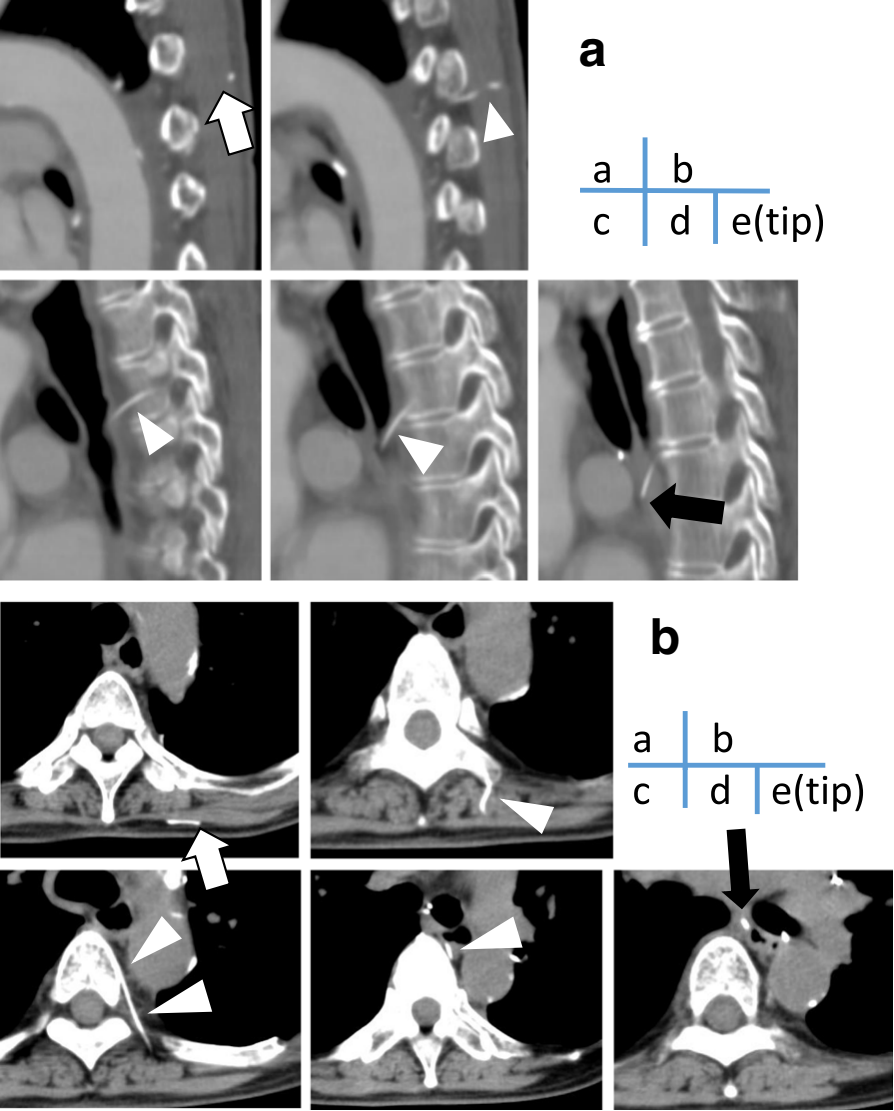
Computed tomography images of the retained catheter fragment (*arrow*). The cut side of the catheter fragment is located at a depth of 6.3 mm from the skin surface (*white arrow*). The tip of the paravertebral catheter (*black arrow*) has been displaced to the posterior mediastinum and is located near the esophagus and trachea, on sagittal views (**a**) and axial views (**b**)

The catheter fragment was not identified on postoperative chest radiography. However, after identification of the fragment on computed tomography (CT), a radiopaque catheter fragment was retrospectively identified near the fifth thoracic vertebral body on the chest radiograph (Fig. 1). The catheter fragment was also visible in the tissue of the back on CT (Fig. 2). Radiologists detected the retained catheter fragment on CT a month and a half after the surgery; however, the attending physician and surgeon did not notice it until this incident.

**Fig. 3:**
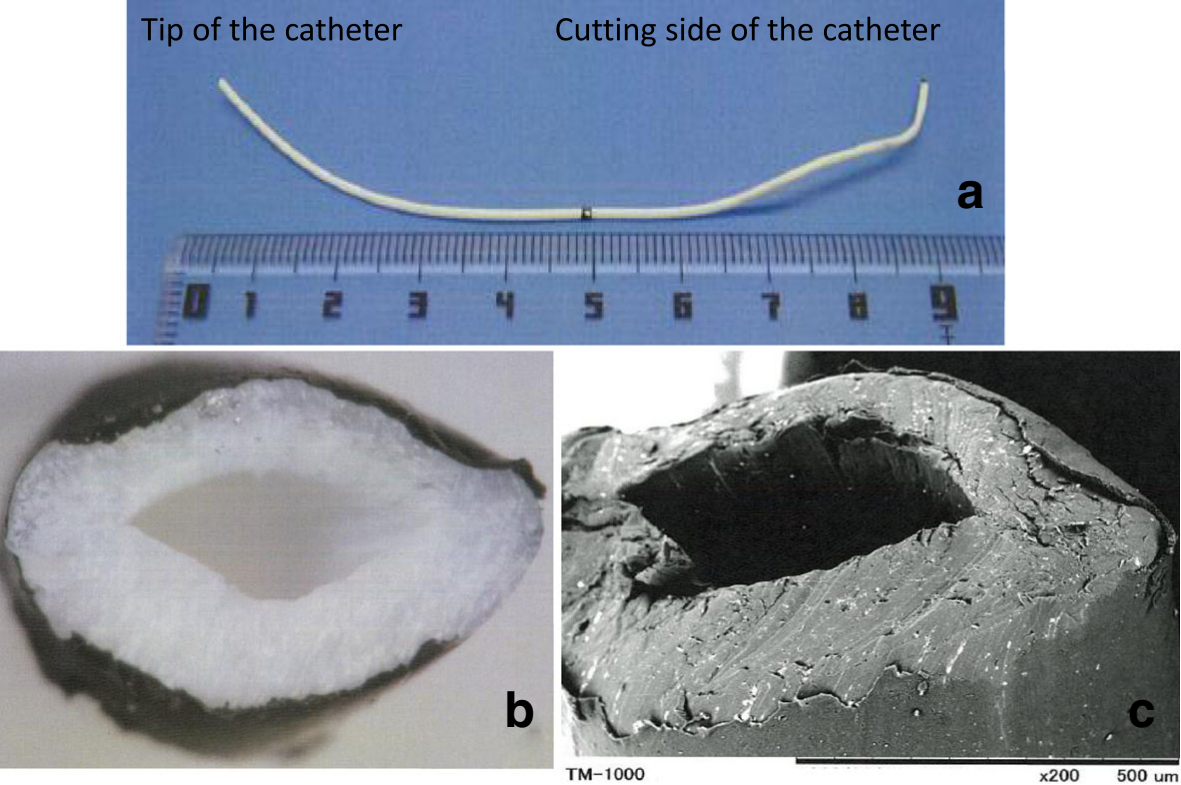
Photograph and transmission electron micrograph of the paravertebral catheter fragment. The catheter was cut 100 mm from the tip (**a**). The catheter was severed without stretching, and the cut surface is slightly crushed, and smooth, on photomacrography (**b**) and transmission electron micrography (**c**)

Two years had passed since insertion of the paravertebral catheter. The patient had neither neurological symptoms nor evidence of infection around the fragment. We considered that pancytopenia as a result of chemotherapy might increase the risk of hematoma or abscess formation around the fragment. Therefore, the catheter fragment was surgically removed. The cut side of the fragment was identified in the muscles of the back and the fragment was completely removed. Analysis of the fragment revealed that the catheter had been severed 100 mm from the tip. The catheter fragment was not stretched, and the cut surface was slightly crushed and smooth (Fig. 3). We hypothesized that the catheter had been cut either by the Tuohy needle or the cutting edge of the needle used to fix it to the skin. The patient was in good general health and experienced no complications after surgical removal of the catheter fragment.

### Discussion

Breakage or shearing of a catheter occurs rarely. Collier [6] showed that the incidence of epidural catheter breakage is approximately 0.002% (1/60,000). There are no well-defined guidelines for the management of catheter fragments retained within the patient. Although surgical removal is recommended for patients with neurological symptoms due to a catheter fragment [6], leaving the fragment in place is considered acceptable in asymptomatic patients [7]. However, neurological symptoms may develop if the retained catheter fragment causes late-onset hematoma [2–5]. A previous report from our hospital [8] describes a patient with neurological symptoms related to late-onset hematoma formation around an epidural catheter fragment. The epidural catheter fragment had been in the epidural space for 18 years. In the present case, the paravertebral catheter fragment was retained in the tissue of the back for 2 years, and the patient experienced no neurological symptoms. If the catheter fragment had been left in the body, chronic inflammation might have occurred around the fragment with resultant vascular fragility. This condition, together with possible coagulopathy as a side effect of chemotherapy, might have resulted in hematoma formation around the fragment. In addition, the retained catheter fragment might have been a source of bacterial infection with resultant abscess formation, particularly if immunosuppression occurred as a result of chemotherapy. Therefore, we decided to remove the catheter fragment surgically.

Breakage of a catheter usually occurs during insertion or removal procedures. In this case, analysis of the fragment revealed that the catheter had been severed by the cutting edge of the Tuohy needle or the suture needle. If a catheter is withdrawn during the insertion procedure, the catheter should not be withdrawn through the Tuohy needle, but be withdrawn together with the Tuohy needle [7]. To prevent movement or removal, once the catheter is placed, it should be fixed to the skin with sutures, surgical tapes, or skin glue. Although secure fixation can be achieved with sutures, the suture needle may damage the catheter. Therefore, to reduce the risk of damage to the catheter, it is necessary to consider the method of catheter fixation. After removal of a catheter, its tip should be checked to ensure that the entire catheter has been completely removed. In this case, it is likely that the tip of the catheter was not checked after catheter removal.

Thoracic paravertebral catheters may be inadvertently inserted into other spaces. Luyet et al. [9] reported the position of the paravertebral catheter tip in human cadavers, inserted with ultrasound-guidance. Of the 36 catheters inserted, the catheter tip was located in the thoracic paravertebral space in 24 (66.6%), the prevertebral or mediastinal space in nine (25%), and the muscle in one (2.7%). No catheters were detected in the epidural space. In our case, the paravertebral catheter was displaced into the posterior mediastinum, and the tip was near the carina and esophagus. The ideal length of a paravertebral catheter and the ideal position of an insertion needle are unknown. Further studies are needed to determine the optimal insertion distance for a paravertebral catheter, and the optimal position of insertion needle into the thoracic paravertebral space.

## Conclusions

This is the first report describing breakage of a paravertebral catheter and retention of the catheter fragment in the posterior mediastinum. The catheter fragment was retained in the patient for 2 years. The patient had neither neurological symptoms nor evidence of infection. The catheter was likely damaged during the insertion procedure. We considered the risk of hematoma and abscess formation around the fragment resulting from chemotherapy-induced coagulopathy or immunosuppression. Therefore, we surgically removed the catheter fragment.

## Abbreviations

CT: Computed tomography
TPVB: Thoracic paravertebral block
